# Factors associated with stroke associated pneumonia among adult stroke patients admitted to university of Gondar hospital, Northwest Ethiopia

**DOI:** 10.1038/s41598-022-14656-2

**Published:** 2022-07-26

**Authors:** Messay Assefa, Abilo Tadesse, Aynishet Adane, Mekonnen Yimer, Melaku Tadesse

**Affiliations:** Department of Internal Medicine, School of Medicine, College of Medicine and Health Sciences, University of Gondar, Gondar, Ethiopia

**Keywords:** Medical research, Neurology

## Abstract

Stroke is the major cause of disability and death in sub-Saharan African countries. The presence and severity of complications play a major role in the outcome of stroke. Stroke associated pneumonia is often noticed post stroke infection that has been linked to an increased risk of hospital mortality, a longer hospital stay and higher healthcare expenses. Report on details of stroke-associated pneumonia has never been documented in countries of sub-Saharan Africa. This study aimed to determine the incidence and risk factors of stroke-associated pneumonia among adult stroke patients in hospital settings, Northwest Ethiopia. The study was undertaken at a stroke care unit, University of Gondar hospital between January 1, 2020 and December 31, 2020. A convenience sampling method was used to recruit study subjects. Relevant clinical history was taken, focused physical examination was done, and brain imaging (CT scan or MRI) was performed to settle the diagnosis of stroke. A modified Centre for Disease Control and Prevention (CDC) criteria was used to diagnose stroke-associated pneumonia. All patients with stroke-associated pneumonia were treated according to the 2016 Infectious Diseases Society of America/American Thoracic Society Clinical Practice Guidelines. The Data were cleaned in Epi Info version 4.6.0.2, and analyzed using SPSS version 26. Variables associated with stroke-associated pneumonia were computed using logistic regression analysis. P value < 0.05 was considered to declare statistical significance. The study comprised a total of 325 adult stroke patients. The mean age of study subjects was 65.2 years (SD ± 15.7). The most prevalent type of stroke was ischemic stroke, which accounted for 68% of all cases. Hemiparesis (94%), facial palsy (87%), and swallowing disturbance (51%) were the frequently noticed neurological findings. Stroke-associated pneumonia complicated 116/325 (36%) of stroke patients. Multi-variate regression analysis revealed that patients who were elderly (age > 75 years) (AOR = 3.910, CI 1.181–12.936, *P* = 0.026), had swallowing disturbance (AOR = 4.656, CI 2.356–9.202, *P*-value < 0.001), epileptic seizures (AOR = 2.678, 95% CI 1.253–5.721, *P*-value < 0.001) and moderate to severe stroke (NIHSS score = 16–21) (AOR = 5.994, 95% CI 2.043–17.585, *P*-value < 0.001) were at risk of developing stroke-associated pneumonia. SAP was a substantial medical complication among stroke patients. Early identification and prompt intervention measures for the identified risk factors might address the burden of SAP.

## Introduction

Stroke is an abrupt onset of focal neurological deficit attributable to a vascular cause^1^. The burden of stroke is high and still increasing in the developing world due to socio-demographic and life style changes^2–5^. Seventy percent of strokes and 80–85% of stroke-related disability and deaths occur in low- and middle-income countries^2–5^. The outcome of stroke mainly depends on the presence and severity of post stroke complications^6^. Stroke associated pneumonia (SAP) complicates 5–44% of all stroke subtypes^7–12^. SAP is defined as pulmonary infections within 7 days of stroke onset^13,14^. SAP was linked to increased risk of hospital mortality, a longer hospital stay and higher healthcare expenses^8,9,15–18^. Hospital-based studies had shown that there are neurological and medical conditions, which would predispose for higher incidence of SAP. Neurological risk factors comprised of altered level of consciousness, severe neurologic deficit, higher NIHSS score, large stroke size, middle cerebral artery stroke, prior stroke, dysphagia, dysarthria/aphasia and cranial nerve palsy^8–12,18–21^. While, medical risk factors consisted of older age, male gender, poor performance status, atrial fibrillation, anemia, hypoalbuminemia, hyperglycemia, endotracheal intubation, nasogastric tube feeding, and comorbidities such as heart failure, diabetes mellitus, and chronic lung disease^8–12,18,19,22,23^. Incidence and predictors of SAP among stroke patients were not reported in countries of sub-Saharan Africa. Hence, this research would fill in the information gap by estimating the magnitude and predictors of SAP among hospitalized stroke patients in the region.

## Methods

### Study setting

The study was conducted at a stroke care unit, University of Gondar hospital between January 1, 2020 and December 31, 2020. The hospital is located in Northwest Ethiopia. It is a tertiary referral and teaching medical institute with a bed capacity of 600, and had a catchment population of 7 million people. The hospital had all the major and minor clinical departments. Hematological tests, biochemical tests, serological tests, and stains and culture of specimens were available in the Laboratory department. The radiology department was functioned with X-ray, Ultrasound, CT scan, and MRI. The study was carried out in department of internal medicine. It had 5 general wards with 110 beds, 1 medical ICU with 12 beds, and 5 outpatient clinics. Other services in the department included MDR-TBC service, dialysis service, endoscopy service, HIV/AIDS care, and chronic illness care. The stroke care unit was within general medical wards, and had 15 beds. It provided health care services for inpatients with stroke. Other medical ward beds could be used as required for admission of stroke patients.

### Study population and study subjects

The study population consisted of stroke patients admitted to the hospital. The study subjects were adult stroke patients admitted to the stroke care unit of the hospital during the study period.

### Inclusion criteria

Patients 18 years or older, who were admitted to stroke care unit with the diagnosis of CT or MRI evidenced stroke during the study period were included in the study.

### Exclusion criteria

Patients, who had no neuroimaging (brain CT scan/MRI), admitted after 7 days of stroke onset, and were unable to give consent or were unable to obtain consent from their caregivers, were excluded from the study.

### Study variables

*Dependent variable* Stroke associated pneumonia.

*Independent variable* 1) Socio-demographic characteristics include age, sex, and residence 2) Clinical characteristics include admission time (in hours) from stroke onset; presenting neurological features; site of brain lesion; stroke subtypes; stroke severity (NIHSS scores) 3) Behavioral factors include cigarette smoking and alcohol intake.

### Sample size and sampling procedure

The sample size was calculated based on a single population proportion formula using 95% confidence level, 5% margin of error, 26% previous estimated proportion of SAP, and 10% for non-response rate. A convenience sampling method was used to recruit 325 study participants^24^.

### Data collection instrument and procedure

A semi structured, pre-tested questionnaire was used to collect the clinical data. It was prepared in English and local language (Amharic) for data collection, and the translation was made by conserving its consistency. Thirty three patients had been involved in pilot research to check for the consistency and reliability of the questionnaire. Relevant clinical history was taken, focused physical examination was done, and brain imaging (CT scan/MRI) was performed to establish the diagnosis of stroke. Complete blood count, urinalysis, and serum biochemical tests including liver function tests, renal function tests, plasma glucose level, serum lipid panel and serum electrolytes were determined to each of the patients. Patients with stroke of cardiac source had ECG (ECG 1200G, YSIP-155, Beijing, China) and 2-D Echocardiography with Doppler evaluation (B/W Digital Ultrasound Scanner, ARI Group, China). Patients with pre-existing lung disease had chest X-ray on admission. Stroke patients were followed daily up to 7 days of stroke onset in the hospital for symptoms or signs of pneumonia such as fever, cough, expectoration, dyspnea, new onset altered mentation, tachypnea, respiratory crackles, bronchial breath sounds and desaturation. Patients with suspected SAP would have chest X-ray. Modified Centre for Disease Control and Prevention (CDC) criteria was used to diagnose SAP^13,14^.

### Stroke care outline in the hospital

All stroke patients admitted to stroke care unit were side positioned, with head of the bed elevated to 30° for those at risk of aspiration or suspected to have increased intracranial hypertension. Side positioning was changed every 2 h. Airway, breathing and circulation were maintained via oral airway or intubation, supplemental oxygen, and crystalloid solutions, respectively. Water swallow test (WST) was done to all patients within 24 h of admission. Nasogastric tube was inserted for patients who had swallowing disturbance. WST was re-assessed weekly, if patients could maintain oral feeding. Oral cavity was cleaned with saline on daily basis. Bladder and bowel care were maintained. Fever was treated with antipyretics, and blood sugar level was controlled at 140–180 mg/dl. Low dose subcutaneous heparin was used for deep vein thrombosis prophylaxis. Thrombolytic therapy was not available to treat acute ischemic stroke. All co-morbidities were managed accordingly. All patients diagnosed to have SAP were treated with intravenous antibiotics.

### Data analysis

The Data were entered into and cleaned in Epi Info version 4.6.0.2 (Epi Info, Atlanta, USA), and were exported into and analyzed in SPSS version 26 (SPSS Inc., Chicago, USA). Frequencies and percentages were used for categorical variables, while mean with standard deviation for continuous variables. The results were shown in frequencies and tables. Logistic regression analysis was applied to identify explanatory variables significantly associated with the occurrence of SAP. Those variables with a *P*-value < 0.25 in the bi-variate analysis were exported to multi-variate analysis. The goodness of fit of the model was judged from the Hosmer–Lemeshow test, and was considered acceptable (*P*-value = 0.69). The results were presented as odds ratio with 95% confidence interval. *P*-value < 0.05 was used to declare significant association^24^.

### Ethical considerations

The Institutional Review Board (IRB) of the College of Medicine and Health Sciences, University of Gondar (25/03/2020; IRB No. 1955/03/2020) had given the ethical approval. The study protocol was performed in accordance with the Declaration of Helsinki. Written informed consent was obtained from the study subjects or their caregivers. All obtained data were treated confidentially. Those patients diagnosed with SAP were taken care of as per the recommendation of the 2016 Clinical Practice Guidelines by the Infectious Diseases Society of America (IDSA) and the American Thoracic Society (ATS)^25^.

### Definition of terms

“Stroke: A focal or global disturbance of cerebral function of sudden onset lasting 24 h or longer, or leading to death with no apparent cause other than that of vascular origin”^1^. Stroke types were classified as ischemic stroke or hemorrhagic stroke based on neuroimaging (CT or MRI) findings according to the International Classification of Disease (ICD)-10 Code.

“Stroke associated pneumonia (SAP) is defined as at least one of the following: Fever (>38°C) with no other recognized cause; leukopenia (<4000 WBC/mm3) or leukocytosis (>12 000 WBC/mm3); altered mental status in an adult ≥70 years old with no other known cause; and at least two of the following: New onset of purulent sputum or change in character of sputum; new onset or worsening cough; dyspnea; tachypnea (respiratory rate>25/min); respiratory crackles, bronchial breath sounds; or worsening gas exchange (eg, O_2_ desaturation [Sp02 ≤ 90%); and chest radiographs with at least one of the following: New or progressive infiltrate, consolidation, or cavitation”.

“Probable SAP: all CDC criteria were met in the absence of diagnostic changes on chest x-ray (or where chest x-ray not undertaken), and there was no alternative explanation or diagnosis”^13,14^.

“Definite SAP: all CDC criteria were met, including diagnostic chest x-ray changes”^13,14^.

Water Swallowing Test (WST): 30 ml water from a cup was swallowed in 5 s. The procedure was reported as failed if there was evidence of coughing, choking, voice change, desaturation (Spo_2_ < 90%) or increased breathlessness^26^.

Stroke Care Unit: An organized in-hospital facility that is entirely devoted to the care of stroke patients. The stroke care unit team consisted of neurologists, internists, medical residents, medical practitioners, medical interns, physiotherapists, and unit nurses.

### Ethics approval and consent to participate

The study was performed in accordance with the Declaration of Helsinki and approved by the Institutional Review Board of the College of Medicine and Health Sciences, University of Gondar (25/03/2020; IRB No. 1955/03/2020). Written informed consents for participation were obtained from study subjects or their caregivers.

### Consent for publication

Not applicable.

## Result

### Socio-demographic characteristics of study participants

A total of 325 adult stroke patients were included in the study. The mean (± SD) age of stroke patients was 65.2 (± 11.7) years. More than half (53%) of study participants were females. Majority (67%) of study subjects were rural residents (Table 1).

**Table 1:** Socio-demographic and behavioral characteristics of adult stroke patients admitted to the Stroke Care Unit, University of Gondar hospital, January 1 to December 31, 2020. (n = 325).

Variables	Frequency (No.)	Percentages (%)
**Age**		
18–44	41	12.6
45–55	41	12.6
56–65	71	21.8
66–75	92	28.3
76 and above	80	24.7
**Gender**		
Male	152	46.8
Female	173	53.2
**Address**		
Urban	106	32.6
Rural	219	67.4
**Alcohol intake**		
Yes	81	24.9
No	244	75.1
**Cigarette smoking**		
Yes	15	4.6
No	310	95.4

### Neurological characteristics of stroke patients

Ischemic stroke was the most common stroke subtype, which accounted for two-thirds of all strokes. Approximately three-fourths of stroke patients were admitted within 24 h of stroke onset. Hemiparesis, facial palsy and dysphagia were the frequently noticed neurological findings. A quarter (26%) of stroke patients had predisposing co-morbidities for SAP, which included heart failure, diabetes, COPD and chronic kidney disease (Table 2).

**Table 2:** Neurological characteristics of adult stroke patients admitted to the Stroke Care Unit, University of Gondar hospital, January 1 to December 31, 2020. (n = 325).

Characteristics	Frequency (No.)	Percentages (%)
**Admission time (hrs) from stroke onset**		
≤ 24 h	238	73.2
24–72 h	41	12.6
Above 72 h	46	14.2
**Stroke subtypes**		
Ischemic	221	68.0
Hemorrhagic	104	32.0
**Location of lesion**		
Cortical	158	48.6
Subcortical	159	48.9
Brain stem	8	2.5
**Presenting neurological features**		
Vomiting	97	29.9
Seizure	49	15.1
Facial weakness	284	87.4
Swallowing disturbance	166	51.1
Hemiparesis	310	95.4
**NIHSS score (stroke severity)**		
1–4 (mild stroke)	63	19.4
5–15 (moderate stroke)	232	71.4
16–21 (moderate to severe stroke)	30	9.2
**Extremity weakness (power grading)**		
0–2/5	222	68.3
3–4/5	88	27.1
5/5	15	4.6

### Clinical and laboratory presentation of patients with stroke associated pneumonia

One-hundred sixteen (36%) stroke patients developed stroke associated pneumonia. Almost all SAP patients had cough, and often complained fever. Two-thirds of SAP patients developed tachypnea and respiratory crackles. Oxygen desaturation (Sp0_2_ < 90%) and leukocytosis were observed in majority of SAP patients (Table 3).

**Table 3:** Clinical and laboratory profile of adult stroke patients with stroke associated pneumonia admitted to Stroke Care Unit, University of Gondar hospital, January 1 to December 31, 2020. (n = 116).

Characteristics	Frequency (No.)	Percentages (%)
**Cough (new onset or worsening)**		
Yes	108	93.1
No	8	6.9
**Fever ≥ 38 °C**		
Yes	76	65.5
No	40	34.5
**Respiratory rate**		
< 25	43	37.1
≥ 25	73	62.9
**Respiratory crackles**		
Yes	72	62.1
No	44	37.9
**Oxygen saturation (Sp0**_**2**_**) %**		
≤ 90	71	61.2
> 90	45	38.8
**WBC (× 10**^**3**^**/µl)**		
≤ 12,000	42	36.2
> 12,000	74	63.8

### Factors associated with stroke associated pneumonia

Explanatory variables as predictors of SAP among stroke patients were determined by logistic regression analysis. Bi-variate analysis revealed that stroke patients, who were elderly (age > 75 years), had dense hemiplegia, facial weakness, epileptic seizures, moderate to severe stroke (NIHSS score = 16–21), and swallowing disturbance were found to be at risk of developing SAP. When variables with P value < 0.25 in bivariate analysis were regressed further for multivariate analysis, older age (> 75 years) (AOR = 3.910, CI 1.181–12.936, *P* = 0.026), epileptic seizures (AOR = 2.678, 95% CI 1.253–5.721, *P*-value < 0.001), moderate to severe stroke (AOR = 5.994, 95% CI 2.043–17.585, *P*-value < 0.001), and swallowing disturbance (AOR = 4.656, CI 2.356–9.202, *P*-value < 0.001) were found to be predictors of SAP (Table 4).

**Table 4:** Bi-variate and multi-variate analyses of factors associated with stroke associated pneumonia among adult stroke patients admitted to Stroke Care Unit, University of Gondar hospital, January 1 to December 31, 2020. (n = 325).

Variables	Stroke associated pneumonia		Crude odds ratio	*P* value	Adjusted odds ratio	*P* value
	Yes	No				
**Age**						
18–44	5	36	1		1	
45–55	14	27	1.267 (0.668–2.332)	0.448	1.220 (0.557–2.673)	0.619
56–65	27	44	1.267 (0.661–2.432)	0.476	1.322 (0.661–2.646)	0.430
66–75	35	57	1.502 (0.696–3.279)	0.310	1.741 (0.651–4.655)	0.269
76 and above	35	45	5.613 (1.990–15.756)	0.010	3.910 (1.181–12.936)	0.026
**Gender**						
Male	58	94	1.223 (0.776–1.928)	0.385		
Female	58	115	1			
**Address**						
Urban	38	68	1			
Rural	78	141	1.010 (0.623–1.639)	0.967		
**Vomiting after onset of stroke**						
Yes	38	59	1.23 (0.750—2.020)	0.393		
No	78	150	1			
**Facial weakness**						
Yes	107	177	2.071 (0.949–4.516)	0.067	1.066 (0.268–4.232)	0.928
No	10	31	1		1	
**Epileptic seizure**						
Yes	28	21	2.84 (1.530–5.290)	< 0.001	2.678 (1.253–5.721)	< 0.001
No	88	188	1		1	
**Location of lesion**						
Cortical	65	93	1		1	
Subcortical	47	112	0.699 (0.169–2.896)	0.621	0.415 (0.057–3.021)	0.385
Brain stem	4	4	1.666 (1.046–2.652)	0.032	1.286 (0.734–2.254)	0.079
**Stroke subtype**						
Ischemic	77	145	0.871 (0.537–1.415)	0.578		
Hemorrhagic	39	64	1			
**NIHSS**						
< 5	12	51	1		1	
5–15	79	153	9.684 (3.570–26.266)	0.015	4.630 (0.959–22.358)	0.056
> 15 (16–21)	25	5	21.250 (6.743–66.963)	< 0.001	5.994 (2.043–17.585)	< 0.001
**Extremity weakness**						
0–2	94	128	2.937 (0.806–10.702)	0.112	1.872 (0.272–12.765)	0.455
3–4	19	69	1.667 (0.503–4.732)	0.095	1.375 (0.597–3.164)	0.522
5	3	12	1		1	
**Water swallowing test**						
Pass	24	135	1		1	
Failed	92	74	6.993 (4.111–11.895)	< 0.001	4.656 (2.356–9.202)	< 0.001

## Discussion

A total of 325 stroke patients were included in the study. The mean age of study participants was 65.2 (± 11.7) years, which was within the African stroke age range (55–67 years)^15–17,27,28^. Africans have a 15-year lower stroke age than people in the developed world^2–5^. Differences in life style, genetic factors, stroke predisposing factors, and population pyramids might be responsible. In this study, one-third (32%) of the stroke subtypes was hemorrhagic, which was within the lower range of African reports. Hemorrhagic stroke accounted for 30–54% of stroke subtypes in Africans, whereas it constituted for 5–10% stroke subtypes in developed world^2,4,15,17^. Evolving urbanization and concomitant social stress, genetic factors, dietary and life style changes, and the high burden of undiagnosed and uncontrolled hypertension could explain the higher proportion of hemorrhagic stroke in Africans^4,5,15,17,28,29^. Three-fourths (73%) of stroke patients were hospitalized within 24 h of stroke onset. Similarly, a Sierra Leonean study revealed that majority (62%) of stroke patients were admitted within 24 h of stroke onset^17^. In a contrary, a Zambian study described that majority (72%) of stroke patients were admitted after 24 h of stroke onset^15^. Another study in Ethiopia had shown that the median time from stroke symptom onset to admission was 48 h^28^. The difference in timing of stroke admissions among sub-Saharan countries could be explained by difference in health seeking behavior, access to a nearby health facility for early referral, and a preference to use alternative traditional treatments. Delay in admission could waste an effective intervention period to minimize subsequent neurological insults^16,27^. Stroke associated pneumonia complicated 36% of stroke patients. There was no any report on incidence of SAP among stroke patients in countries of sub-Saharan Africa. The incidence of SAPin Asian countries was 11–22%, though it was only 5–7% in the Western world^8–11^. A difference in the excellence of health care services might be a reasonable justification. On multi-variate regression analysis, stroke patients, who were elderly, had swallowing disturbance, epileptic seizures, and moderate to severe stroke were at risk of developing SAP. Patients older than 75 years were four folds more at risk of developing SAP as compared to younger age groups (AOR = 3.910, CI 1.181–12.936, *P* = 0.026). The increased prevalence of SAP in elderly might be due to co-existing swallowing difficulties, immunosuppression, co-morbidities and frailty^9–11,18,19,21,22^. The odds of developing SAPwere four and a half times higher in stroke patients who had swallowing disturbance as compared to those who did not (AOR = 4.656, CI 2.356–9.202, *P*-value < 0.001). Swallowing disturbance was reported in 37–78% of stroke patients, and was responsible for more than a threefold increased risk of SAP. Swallowing disturbance frequently manifested with disordered lingual and pharyngeal movements, and could cause gross aspiration of food or liquids. Confirmed aspiration was accounted for more than a tenfold increase in SAP^8,11,12,20,26^. A nasogastric (NG) tube was inserted for the feeding of stroke patients who had swallowing disturbance. NG tube use often offered protection of the airway. However, it might predispose for SAP as being a site for bacterial biofilm formation**.** In addition, it could result in the reflux of bacterial laden gastro-esophageal secretions^12,20,26^. Stroke patients who had epileptic seizures were two and half times more likely to develop SAPas compared to those who did not (AOR = 2.678, 95% CI 1.253–5.721, *P*-value < 0.001). Impaired airway control and aspiration of bacterial rich oro-pharyngeal secretions during seizure episodes might be a plausible explanation^8,18,19,22,23^. Patients who had moderate to severe stroke (NIHSS scores = 16–21) had a six fold greater risk of developing SAP than those who had mild stroke (AOR = 5.994, 95% CI 2.043–17.585, *P*-value < 0.001). Various studies had shown that stroke patients with marked brain injuries were unable to protect their airway and experienced gross aspiration. In addition, stroke patients with marked brain injury might experience immunosuppression, which could be explained by augmented sympathetic drive and high stress hormone level^9–11,18,21–23^.

### Limitation of the study

Hospitalized patients, severely ill study candidates, might be recruited as study subjects. Only stroke patients who had undergone neuroimaging (CT scan/MRI) were included in the study. The non-probability sampling method was used to recruit study subjects. All these clinical conditions might introduce selection bias.

### Conclusions

SAP was a substantial medical complication in stroke patients. Stroke patients, who were elderly, had swallowing disturbance, epileptic seizures, and moderate to severe stroke were at risk of developing SAP.

### Recommendation

Early identification and prompt intervention measures for the identified risk factors might address the burden of SAP.

## Data Availability

All data generated and analyzed were included in this research article.

## Abbreviations

AOR: Adjusted odds ratio
ATS: American thoracic society
COR: Crude odds ratio
COPD: Chronic obstructive pulmonary disease
CXR: Chest-X-Ray
CI: Confidence interval
CKD: Chronic kidney disease
CDC: Centers for disease control and prevention
IDSA: Infectious Disease Society of America
NIHSS: National institute of health stroke scale
PO: Per Os
SAP: Stroke associated pneumonia
SD: Standard deviation
WBC: White blood cells
